# Reviewers Acknowledgement

**DOI:** 10.1002/1878-0261.13363

**Published:** 2023-01-04

The Editors of *Molecular Oncology* would like to thank all referees who have given their time and expertise to review articles submitted for publication in Volume 16. *Molecular Oncology* heavily relies on reviewers' careful assessment, constructive criticism, and timely response to ensure both quality and fast turnaround times.

The names of these reviewers are listed below; we apologize if we have inadvertently omitted anyone.

Adusumilli Prasad

Ahmad Mohammad

Ahmad Imran

Ahmed Wareed

Alghamdi Mohammed

Ali Azhar Bin

Amat Ramon

Amati Bruno

Ammanamanchi Sudhakar

Andersen Claus

Anderson Mary Ann

Angelastro James Michael

Appella Ettore

Aran Veronica

Arguelles Sandro

Arteaga Maria‐Francisca

Arulananda Surein

Arvidsson Per

Ashktorab Hassan

Astanina Elena

Autexier Chantal

Ávila Zaragozá Matías Antonio

Avril Tony

Avsar Timucin

Babačić Haris

Baird Duncan M.

Baniahmad Aria

Bartelt Alexander

Barthet Valentin

Bartoszewski Rafał

Basu Sujit

Basu Devraj

Behrens Jürgen

Beltrán‐Anaya Fredy Omar

Berge Viktor

Berger Frédérique

Bernasconi Michele

Bertagnolo Valeria

Bhak Jong

Bharadwaj Alamelu G.

Biedermann Tilo

Biffi Stefania

Binderup Tina

Blanco Heredia Juan

Blandino Giovanni

Blomme Arnaud

Blum Yuna

Boeri Mattia

Bossis Guillaume

Boyer Laurent

Brenet Fabienne

Bridgewater John

Brunner Cornelia

Buck‐Koehntop Bethany A.

Bussolino Federico

Bustelo Xosé R.

Cabel Luc

Cabot Myles

Cagan Alex

Cai Hao

Cai Guoping

Cai Zhixiong

Camero Simona

Campello Silvia

Cani Andi

Canis Martin

Cao Minghui

Cappello Paola

Caraglia Michele

Carausu Marcela

Carbonell Caterina

Cardaci Simone

Carmona Pedro

Carnero Amancio

Carreras‐Puigvert Jordi

Carver Brett S.

Castaño Justo P.

Castegna Alessandra

Cervera Eduardo

Chan Chang S.

Chandra Dhyan

Chandra Joya

Chang An‐Chen

Chang Guoqiang

Chang Wen‐Wei

Chapman Jessica

Chatsirisupachai Kasit

Cheema Amrita

Cheli Yann

Chen Chen

Chen Ken

Chen Ching‐Hsien

Chen Liangyuan

Chern Edward

Chien Wenwen

Choudhary Bibha

Christensen Emil

Chu Simon

Cimini Daniela

Clarke Robert

Clatot Florian

Clynes Martin

Cockman Matthew

Coffelt Seth

Cohen David S.

Cong Yu‐Sheng

Conklin Douglas S.

Copland Mhairi

Corujo David

Costa Valerio

Costa‐Silva Bruno

Cotelle Philippe

Cros Jerome

Cui Xiaojiang

Cukierman Edna

Culig Zoran

Czyzyk‐Krzeska Maria

da Silva Edaise M.

D'Agostino Vito

D'Alfonso Timothy

Dall Genevieve

Dall'Olio Fabio

Damia Giovanna

D'Angiolella Vincenzo

Daniel Juliet

Davidson Ben

De Pradip

De Bortoli Michele

de Cárcer Guillermo

Deaglio Silvia

Dean Michael

Dejardin Emanuel

Del Sal Giannino

Deng Jinhai

Dequiedt Franck

Derks Sarah

Deshpande Ravindra

Deutsch Eric

Diaz‐Garcia Elena

Diefenbacher Markus E.

Dimas Konstantinos

Dimopoulos Konstantinos

Ding Ling‐Wen

Djouder Nabil

Dopeso Higinio

Dou Zhixun

Drosten Matthias

Durocher Daniel

Ebinger Martin

Edeline Julien

Eferl Robert

Ehata Shogo

Eilers Martin

Eiring Anna

Ekman Simon

Eldering Eric

Enokida Hideki

Enroth Stefan

Ernberg Ingemar

Espinet Elisa

Eugenin Eliseo

Eyal Eran

Farace Francoise

Fazio Grazia

Felley‐Bosco Emanuela

Felli Maria Pia

Feng Hugang

Flamini Marina Inés

Fluegen Georg

Fortunato Orazio

Francavilla Chiara

Frezza Christian

Friboulet Luc

Frigola Joan

Fujita Naoki

Galibert Marie‐Dominique

Galindo Rene

Galli Giorgio

Gammage Payam

Ganapathy Vadivel

Garcia Maria J.

García‐Castillo Jesús

García de Herreros Antonio

García Martín Santiago

Garcia Silva Susana

Gazzaniga Paola

Gebauer Niklas

Geiger Harmut

Germain Doris

Gillespie David

Gilmore Andrew P.

Giraldo Rafael

Gires Olivier

Goenka Anshika

Goergens Andre

Goodwin Jon

Gorman Adrienne

Goto Akiteru

Grana Xavier

Green Darrell

Grillier‐Vuissoz Isabelle

Grumati Paolo

Guerra M. Carmen

Guil Sonia

Guittaut Michaël

Guo Jessie Yanxiang

Guo Siqi

Guttery David

Haakensen Vilde Drageset

Habringer Stefan

Hammond Ester

Han Zheng

Han Weidong

Hansen Torben F

Haricharan Svasti

Harlepp Sebastien

Hayward Simon

Healey Graham F.

Heinzel Susanne

Heitzer Ellen

Herbein Georges

Hermeking Heiko

Hernandez Fernaud Juan Ramon

Hess Jochen

Hickey Peter

Hiltermann Jeroen

Hofman Paul

Holding Andrew N.

Hollande Frederic

Holland‐Letz Tim

Hou Jian

Hsiao Michael

Huang Wei‐Chien

Huang Jian

Huang Danny

Huber Kilian

Hühn Daniela

Humphries Jonathan

Hur Man‐Wook

Ibáñez‐Costa Alejandro

Ishov Alexander

Itoh Shinji

Ivanov Andrei

Janke Florian

Janssen Klaus‐Peter

Janz Siegfried

Jardim Denis L.

Jenab Mazda

Jimenez Connie R.

Jones Rheinallt M.

Kaldis Philipp

Kalvakolanu Dhan V.

Kaplan Tommy

Karteris Emmanouil

Katanaev Vladimir

Kenny Paraic

Kermorgant Stephanie

Ketola Kirsi

Khan Md. Wasim

Khrapko Konstantin

Kim Nam Deuk

Kim Sarah

Kim Cheorl‐Ho

Kirschner Michaela Barbara

Klinakis Apostolos

Ko Eric

Kodous Ahmad

Koinuma Daizo

Koizume Shiro

Komatsu Masaaki

Kosnopfel Corinna

Kumari Sonam

Kuznetsov Vladimir A.

Kypta Robert

Laajala Teemu Daniel

Ladomery Michael R.

Lal Ashish

Lampe Paul D.

Lamy Pierre‐Jean

Lancaster Graeme

Landero‐Huerta Daniel

Lathia Justin Durla

Latonen Leena

Lawlor Rita T.

Lax Sigurd

Le Romancer Muriel

Lecona Emilio

Lee Terence K. W.

Lee Jenny

Lemonnier Francois

León Javier

Leszczynska Katarzyna

Levi‐Schaffer Fransesca

Lewis Robert

Li Yulin

Li Baiwen

Li Kang

Li Sha

Li Hui

Lianidou Evi

Liao Tsai‐Tsen

Liao Mingzhi

Liesa Marc

Lietha Daniel

Lignitto Luca

Lin Hao

Lin Peter P.

Lin Cheng‐Wei

Lindner John

Lingjaerde Ole Christian

List Karin

Liu Xue‐Song

Liu Yanhong

Liu Zhonghua

Liu Yunpeng

Liu Bei

Lofgren Kristopher

Lohinai Zoltan

Lombard David

Long Weiwen

Looijenga Leendert H. J.

Lopez‐Beltran Antonio

Lord Chris

Lorenz Susanne

Lorenzo Montanaro

Lu Yi

Lumniczky Katalin

Lund‐Iversen Marius

Luo Guanghong

Luo Qicong

Madeddu Roberto

Madureira de Oliveira Diêgo

Maggiolini Marcello

Malumbres Marcos

Manavalan Balachandran

Mandal Mahitosh

Marchio Caterina

Martinelli Erika

Martinez‐Barbera Juan Pedro

Martínez‐Fernández Mónica

Martinez‐Jimenez Francisco

Marusyk Andryi

Masuda Muneyuki

Matheu Ander

Matikas Alexios

Maya‐Mendoza Apolinar

Mayoh Chelsea

Mayor‐Ruiz Cristina

Mazumder Aloran

McNeish Iain

Mehta Isha D.

Melo Sónia A.

Mendes Marta

Mereu Elisabetta

Meyer Lüder‐Hinrich

Michelucci Alessandro

Micke Patrick

Mikheev Andrei M.

Milpied Pierre

Mineo Marco

Mishra Avanish

Misra Jyoti

Miyazawa Keiji

Miyazono Kohei

Mohammad Ramzi M.

Mohar Alejandro

Molina‐Vila Miguel A.

Momeni‐Boroujeni Amir

Moreaux Jerome

Morel Etienne

Moreno de Alboran Ignacio

Morgan Iain

Moser Tina

Mosimann Christian

Moustakas Aristidis

Munoz‐Espin Daniel

Nagai Mariaaparecida

Nagy László

Narita Masashi

Nassour Joe

Nastaly Paulina

Nemenoff Raphael

Neubauer Hans

Neuhöfer Patrick

Neureiter Daniel

Nowak Dawid G.

Ntwasa Monde

O'Hagan Heather M.

Ohtani Naoko

Øjlert Åsa

Ollilla Saara

Omelchenko Tatiana

o'Neill Eric

Oppelt Jan

Ou Sai‐Hong I.

Ozcan Sabire

Pagano Michele

Pardini Barbara

Pardo Oliver

Parisse Pietro

Passaniti Antonino

Pateras Ioannis

Paul Thomas

Pecqueur Claire

Pei Xin

Penzo Marianna

Perez de Castro Ignacio

Perez‐Plasencia Carlos

Perretti Mauro

Pertz Olivier

Peschiaroli Angelo

Petronini Pier Giorgio

Peuhu Emilia

Pfeifer Bastian

Phillips Joanna J.

Piaggio Giulia

Pichler Martin

Pierga Jean‐Yves

Pierobon Mariaelena

Pietras Kristian

Pigazzi Martina

Pinho Salomé

Plymate Stephen R.

Po Agnese

Poh Ashleigh

Pol Jonathan

Porta Chiara

Powell Michael Duane

Puissant Alexandre

Radlwimmer Bernhard

Raha Suchismita

Ramachandran Srinivas

Ramadan Kristijan

Rampazzo Chiara

Rantakari Pia

Ratajewski Marcin

Ratnacaram Chandrahas Koumar

Rayala Suresh

Rehm Markus

Reisman David

Remon Jordi

Renault Shufang

Rexer Brent N.

Ribatti Domenico

Ricchetti Miria

Ricciardelli Carmela

Riethdorf Sabine

Rimassa Lorenza

Risbridger Gail P.

Rivas Carmen

Rocheleau Christian E.

Röcken Christoph

Rodriguez‐Aguayo Cristian

Rodriguez‐Henche Nieves

Roesch Alexander

Rohlin Anna

Rolfo Chrisitan

Romero Atocha

Rosell Rafael

Rota Rosella

Rotblat Barak

Ruess Dietrich A.

Ryan Anderson

Saba Julie

Sabatier Renaud

Saeed Faisal

Safonov Anton

Sahali Dil

Sakamoto Shuichi

Salgia Ravi

Samuel Michael

Sana Jiri

Sandahl Sørensen Boe

Sandulache Vlad C.

Sanjay Kumar Singh

Santamaria David

Saretzki Gabriele Christine

Sarungbam Judy

Sasaki Yasushi

Saucier Caroline

Sayan Emre

Scherer Florian

Schettini Francesco

Schewe Denis M.

Schilling Oliver

Schorle Hubert

Schueller Ulrich

Schuuring Ed

Seewaldt Victoria L.

Segui Bruno

Sekido Yoshitaka

Senapati Dhirodatta

Sengupta Surojeet

Sevenich Lisa

Sexton‐Oates Alexandra

Seymour Leonard

Shao Zengwu

Sharma Aarushi

Shaw Andrey

Shaykhiev Renat

Shen Minhong

Shen Lunda

Shi De‐Li

Shimoda Masafumi

Sijts Alice J. A. M.

Skånland Sigrid

Smeland Sigbjørn

Smirnov Artem

Somyajit Kumar

Sprick Martin R.

Spruck Chuck

Stockhammer Paul

Stoecklein Nikolas H.

Strahl Sabine

Sukocheva Olga

Sukumar Saraswati

Sültmann Holger

Sun Qian

Supek Fran

Szabo Roman

Szymanski de Toledo Marcelo

Tabernero Arantxa

Tagliabue Elda

Takahashi Akiko

Taly Valerie

Tang Hailin

Tasken Kjetil

Teixeira Samuel

Terada Lance S.

Thai Alesha

Thijssen Rachel

Tie Jeanne

Tiensuu Janson Eva

Timpson Paul

Torres Adrian Gabriel

Triola Gemma

Tripathi Satyendra Chandra

Tukachinsky Hanna

Tulasne David

Turashvili Gulisa

Tyagi Abhishek

Ulasov Ilya

Vaags Andrea

Vadlamudi Ratna K.

Vallejo Alejandro

van den Broek Daan A.

van der Stok Eric P.

van Kempen Léon C.

van Ness Brian

Van Tine Brian

Vandenbempt Isabelle

Vecchi Lara

Vecera Marek

Venere Monica

Vidal‐Barull Joana

Vietri Maria

Vijg Jan

Vlahou Antonia

Vlashi Erina

Vleminckx Kris

Volpi Emanuela

von Bubnoff Nikolas

Vouret‐Craviari Valerie

Wallace Nicholas

Wang Tianzhen

Wang Xiaosong

Wang Yaohe

Ward Douglas

Waring Mike

Washburn Michael

Weber Martin

Wei Wenyi

Wei Daoyan

Weiren Luo

Wellens Judith

Welsh Joshua

Werner Bonnita

Westermarck Jukka

Weyemi Urbain

Wikman Harriet

Williams Owen

Willis Anne

Windhorst Sabine

Witze Eric S.

Woloszynska Anna

Worst Thomas S.

Wurdak Heiko

Xia Shu‐Jie

Xie Jindong

Xu Haiyan

Xu Naihan

Xue Xiang

Yaeger Rona

Yamamoto Yusuke

Yin Shanshan

Yin Ling

Yochum Gregory

Yu Seong‐Woon

YuanYuan Li

Zaman Guido J. R.

Zawacka‐Pankau Joanna

Zdzalik‐Bielecka Daria

Zhan Qimin

Zhang Hanwen

Zhang Lei

Zhang Rugang

Zhang Pengju

Zhang Naixia

Zhang Qiong

Zhang Wenli

Zhao Zhi

Zhavoronkov Alex

Zhong Jun

Zhou Binhua P.

Zhou Meng

Zhu Huayu

Zhu Honglin

Zhuang Changshui

Zlotta Alexandre R.

Zoeller Margot

Zuna Jan

